# A Wearable Acoustic-Bluetooth Dual Model Communication-Based Real-Time Heart Rate Monitoring and Ranging System for Swimmers

**DOI:** 10.3390/s26103074

**Published:** 2026-05-13

**Authors:** Pingao Huang, Zhihong Xu, Tianzhan Huang, Zhenhua Chen, Junrong Hu, Hui Wang

**Affiliations:** 1 School of Electronic Engineering and Automation, Guilin University of Electronic Technology, Guilin 541004, China; 2 Shenzhen RunYiTaiYi Technology Co., Ltd., Shenzhen 518055, China; 3 CAS Key Laboratory of Human-Machine Intelligence-Synergy Systems, Shenzhen Institutes of Advanced Technology, Chinese Academy of Sciences (CAS), Shenzhen 518055, China; 4 Key Laboratory of Intelligence Integrated Automation in Guangxi Universities, Guilin 541004, China

**Keywords:** underwater acoustic communication, wearable, low power consumption, narrow-pulse OOK, real-time heart rate monitoring

## Abstract

Underwater communication devices typically suffer from large size and high power consumption, which pose significant challenges for real-time monitoring of swimmers’ heart rate and distance. To tackle these challenges, this study successfully developed a wearable acoustic-Bluetooth dual model communication-based real-time heart rate monitoring and ranging system (WARM) for swimmers by implementing an integrated miniaturized acoustic transducer design, narrow-pulse OOK modulation, and acoustic multipath interference suppression techniques. The final self-developed system measures 47 mm × 36 mm × 18 mm and weighs 54 g. Six swimming volunteers were recruited to conduct underwater real-time heart rate monitoring and distance measurement experiments for performance evaluation of this self-developed system. Experimental results demonstrate that within an effective communication range of 2500 cm, the system achieved an average transmission power consumption of 52–58 mW, a frame loss rate of only 1.1%, and a mode-switching time of 1–2 s between the underwater acoustic and Bluetooth transmissions. In addition, the system enabled real-time heart rate monitoring and underwater ranging, with an average ranging error below 50 cm. These results verify the reliability and stability of the proposed system and provide a useful reference for the design and application of wearable underwater communication systems.

## 1. Introduction

Heart rate is a crucial physiological indicator for evaluating human health. During activities like diving and swimming, real-time heart rate monitoring not only helps prevent safety risks but also provides a reference for improving training performance [1]. However, underwater heart rate monitoring faces several challenges, such as waterproofing and electrode attachment, with underwater wireless communication being one of the most critical issues. Currently, underwater wireless communication primarily relies on three methods: radio frequency, optical, and acoustic transmission [2]. These wireless signals are susceptible to factors such as water density, temperature, salinity, and multipath interference, resulting in unstable transmission and low data rates [3].

Because water significantly attenuates electromagnetic waves, the loss of radio frequency signals increases exponentially as frequency rises [4]. Whether using ultra-high frequency, very high frequency, or high-frequency bands, reliable underwater communication becomes difficult once the distance exceeds 10 m [5]. This problem is especially severe in standard unlicensed bands, such as 2.4 GHz, where water causes even greater signal attenuation [6]. Underwater optical communication, with its broad bandwidth and high data rates, is a promising alternative that has attracted significant interest [7,8]. However, optical waves struggle not only to penetrate the air–water interface effectively but also have transmission performance that largely depends on water turbidity [9,10], creating significant challenges for the system design. In contrast, acoustic waves have low attenuation coefficients and strong penetration capabilities in water, allowing for excellent long-distance propagation. These features provide acoustic communication with clear advantages in underwater applications, making it the preferred choice for many systems [5], particularly in scenarios where high data rates are not essential [11]. Nevertheless, applications such as real-time underwater heart rate monitoring require additional features, including a compact size, lightweight design, low power consumption, and ease of wear.

In recent years, the development of underwater wireless sensor networks (UWSN) has driven increasing interest in low-power and miniaturized underwater acoustic communication devices. Jeon et al. developed an ultra-compact acoustic modem that achieves a system power consumption of approximately 2.9 W when powered by a 14.8 V lithium-ion battery, while supporting a communication range of up to 40 m. This work provides a foundation for further advances in the underwater acoustic communication technologies [12]. Won et al. subsequently proposed a miniature underwater acoustic modem based on an ARM Cortex-M3 microcontroller, achieving a communication range of 70 m with a power consumption of approximately 4.5 W [13]. Van Kleunen et al. further developed the Proteus II integrated underwater sensor node platform, which consumes about 8 W during transmission and 300 mW in the receiving state. Under low-duty-cycle communication conditions, the node can operate continuously for approximately one month, providing valuable insights for the design of low-power underwater devices [14]. Mayberry et al. also introduced BlueBuzz, which supports communication distances of up to 220 m, with a receiving power consumption of approximately 2.2 W and a transmission power ranging from 2.4 to 30 W, offering increased flexibility and broader application scenarios for underwater acoustic communication [15]. Campagnaro et al. proposed a software-defined underwater acoustic modem that reduces the transmission power to approximately 1.12 W through a low-power acoustic transmission design while maintaining stable communication over a distance of about 80 m, achieving a favorable balance between energy efficiency and communication performance [16]. In addition, Xu Lijun (2021) and Zou S. (2025) developed a full-ocean-depth acoustic communication system and an acoustic underwater glider, respectively. Both systems demonstrate good stability and low bit error rates in long-distance communication; however, their designs are difficult to adapt to wearable applications [17,18].

In the field of underwater heart-rate health monitoring, Menon et al. proposed a diver remote health monitoring system architecture based on underwater acoustic communication. The system analyzes physiological parameters such as heart rate in real time and triggers acoustic data transmission when abnormal conditions are detected, thereby reducing communication energy consumption [19]. Subsequently, Bube et al. proposed a wearable freediving computer system, which enables data interaction between divers through underwater acoustic communication, with a transmission range of approximately 200 m, an average power consumption of about 2.6 W for its acoustic module, and a continuous operation time of 3–4 h for the system [20]. Furthermore, Xiao ZK et al. designed a real-time physiological signal monitoring device for underwater operation safety. Using PPG sensors to collect the divers’ heart rate, the device achieves real-time underwater monitoring at a 100 m communication distance through underwater acoustic communication, with an acoustic transmission power of approximately 4.8 W [21].

In summary, existing underwater physiological signal monitoring systems have made significant progress in communication range and data transmission capability. However, several limitations remain, including high transmission power consumption, large device size, and high system cost, which restrict their applicability in wearable underwater monitoring scenarios. Table 1 compares the advantages and disadvantages of existing underwater monitoring and communication systems with our proposed system. To address these limitations, this paper proposes a wearable amphibious acoustic-Bluetooth real-time heart rate monitoring and ranging system. The system is compact and low-power and can provide omnidirectional real-time heart rate monitoring and distance measurement within a 25 m range. The system provides an effective technical solution for safety monitoring of underwater athletes and underwater swimming training.

The remaining parts are structured as follows: Section 2 explains the system architecture and design methodologies. Section 3 demonstrates the effectiveness of the system’s application with evaluation results from four experiments. Section 4 discusses the findings of the article. Finally, Section 5 offers the conclusions.

## 2. Materials and Methods

### 2.1. The Architecture of WARM

**Figure 1:**
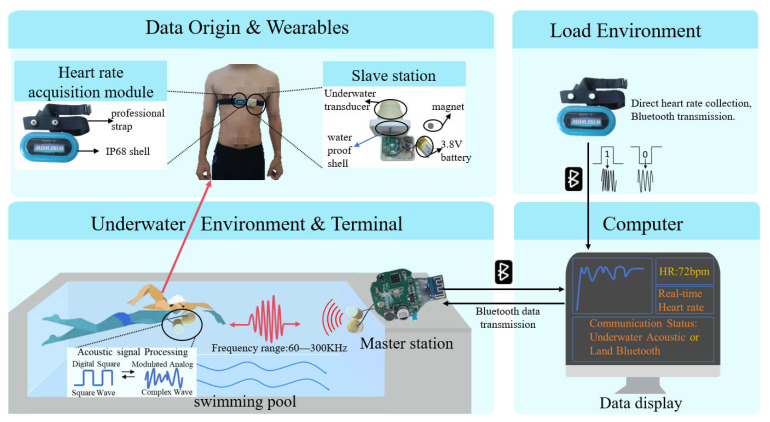
The system block diagram of WARM. Terrestrial heart rate data are transmitted to a computer via Bluetooth; underwater heart rate data are acquired by the slave station, transmitted to the master station, and then sent to a computer.

The system consists of a master station, a slave station, a heart rate acquisition module, and a computer (Figure 1). In a terrestrial setting, heart rate data are transmitted directly to the computer via Bluetooth. Underwater, the computer sends the control commands to the master station, which encodes the data into an acoustic protocol and transmits it to the slave station via an acoustic transducer. The slave station demodulates the signal and returns stored heart rate data to the master station through the same channel. The master station then processes the signal and forwards the data to the computer via Bluetooth. The computer displays real-time heart rate and swimming distance to provide physiological feedback for safety monitoring. To ensure a reliable transmission, the heart rate module and slave station must remain in close proximity to reduce signal attenuation and interference. This study focuses on underwater communication, as terrestrial transmission is relatively straightforward.

### 2.2. The Architecture of the Master and Slave Station

**Figure 2:**
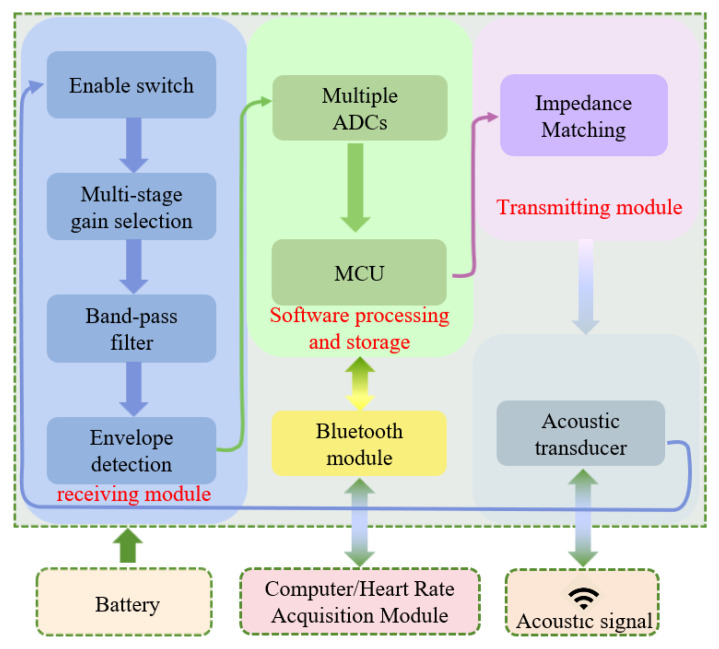
The structure diagram of the master and slave stations. Including the transmitter module, the receiver module, and the signal processing and storage.

The structural block diagram of the master and slave stations is shown in Figure 2. Although their software and hardware architectures are identical, their external designs and power switches vary due to differences in their application circumstances.

#### 2.2.1. Miniaturization and Low-Power Hardware Design

This master and slave station primarily consists of three core circuits: the transmitter module, the receiver module, and the signal processing and storage. Each circuit module adopts a power-off design during non-working hours to reduce system power consumption. The main design approach is described as follows.

**Figure 3:**
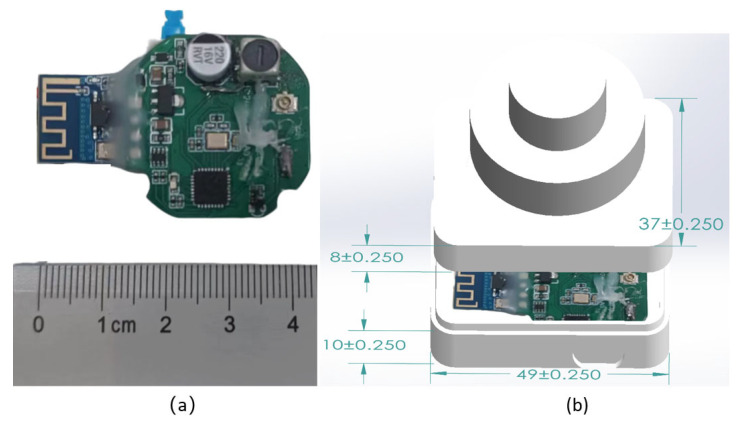
System hardware prototype and slave station assembly drawing. (**a**) Physical hardware prototype of the master and slave stations; (**b**) 3D assembly model of the slave station designed via SolidWorks 2024.

(1) The transmitter module utilizes impedance matching and gain optimization, along with pulse width modulation (PWM) technology and a custom 1:20 transformer to propel the hydroacoustic transducer at high voltage, guaranteeing broad coverage and a reliable communication connection. The low-power MCUs and SOT-23 packaged NMOS transistors are adopted to reduce on-resistance, thereby achieving a low-power hardware design.

(2) The signal receiving module includes an enable switch, multi-stage gain control, band-pass filtering, and envelope detection. The MCU controls the power supplied to the hardware by using the enable switches to reduce the average power consumption. The multi-stage gain control employs a series voltage division method. The band-pass filter isolates the target frequency band and aligns with the transducer’s operating frequency. The envelope detection circuit transforms the AC signal into a unilateral envelope signal, facilitating easier processing.

(3) The signal processing and storage section consists of the MCU and its built-in multi-channel A/D converters. After envelope detection, the processed signal enters the A/D conversion chain, allowing the optimal signal to be fed into the MCU for accurate decoding.

(4) The acoustic transducer is made of piezoelectric ceramic material and features an omnidirectional communication design. Compared to traditional ceramic energy transducers, it offers excellent performance in terms of directivity, transmitting voltage response, and maximum source level [22]. In PCB design, miniaturized components and a four-sided board are employed to enable a high-density layout and a compact form factor. The overall hardware dimensions are 41 mm × 30 mm × 1.6 mm, as shown in Figure 3a. In terms of the structural design, to ensure wearability, the slave station housing uses a compact box-shaped design measuring 47 mm × 36 mm × 18 mm. The structure features upper and lower sections, with the junction bonded together with B7000 waterproof glue to ensure maximum waterproof effectiveness. Meanwhile, a magnetic control switch is employed as the main power control method, and a wireless charging design is employed to ensure reliable operation of the system during underwater communication. The assembly of the slave station is shown in Figure 3b.

#### 2.2.2. Low-Power Software Design

**Figure 4:**
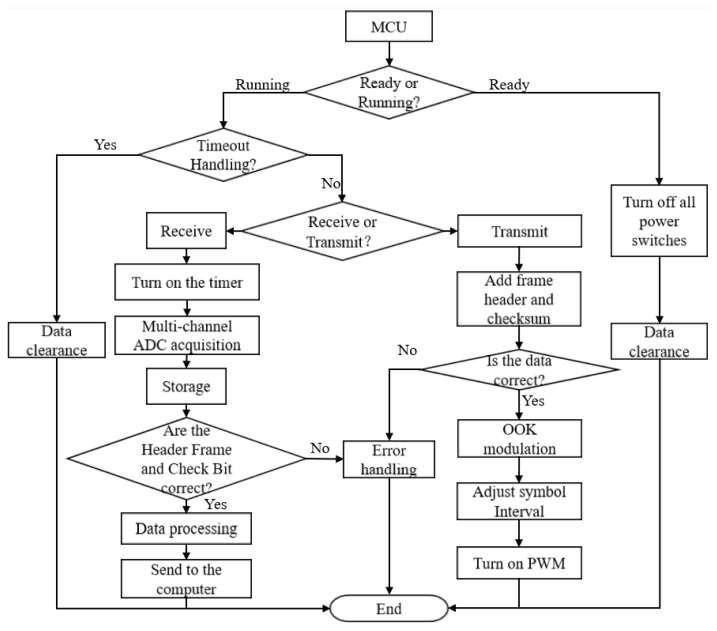
Algorithm flowchart.

Figure 4 illustrates the comprehensive algorithm flowchart of the transceiver, which is principally segmented into transmitting and receiving components. The transmission and reception of communication mechanisms are designed with low power consumption.

1. Transmission components of communication

**Figure 5:**
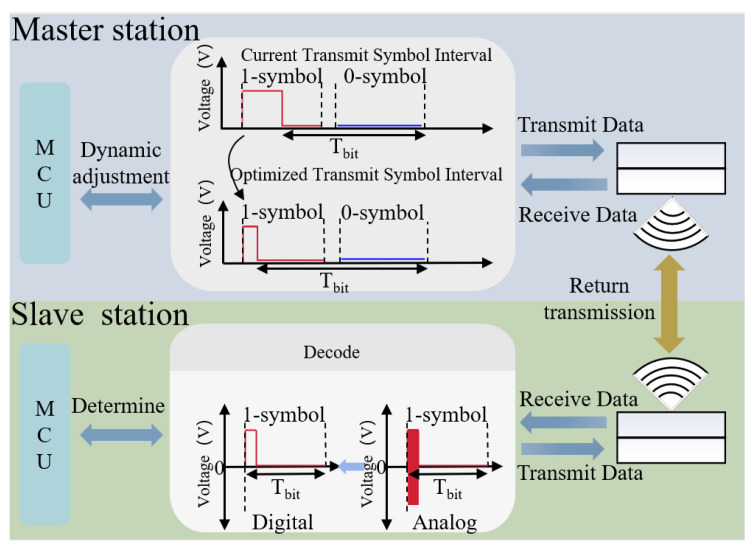
Block diagram of the dynamic symbol interval adjustment strategy for multipath interference mitigation and transmit power reduction.

In underwater communications, when the transmission distance exceeds the water depth, acoustic signals are prone to temporal spreading and multipath interference, which degrade communication performance. To address this issue, a dynamic symbol interval adjustment strategy is adopted. The master station establishes the duration of the symbol and the duration of the high level within a 1-code element. The symbol interval and codeword width are constantly modified according to the quantity of signals fed back to the slave station to maintain signal stability and validity, while efficiently mitigating multipath interference and reducing transmit power. The corresponding block diagram is shown in Figure 5.

The detailed transmit process is illustrated in Figure 4. Initially, attach a frame header and incorporate an XOR parity check into the unprocessed data. Subsequently, make the binary amplitude-keyed (OOK) modulation, modify the symbol interval, and ultimately initiate the PWM transmission.

2. Reception components of communication

The system needs to address signal delays and attenuation resulting from multipath interference, as well as the impact of the time-varying characteristics of the underwater channel on the reliability of signal transmission.

To address these challenges, the transceiver uses a high-frequency sampling method with timed triggering. Upon identifying a designated pre-wake frame header signal, the MCU will promptly awaken and initiate timer sampling to guarantee data precision and dependability. During the signal discrimination phase, the threshold is modified in real time according to channel estimates, dynamically calibrating the comparator threshold using the current and the two prior signal amplitude measurements. The specific formula is as follows.

(1) $$T_{new}=\min(\max(\alpha \times \frac{\sum_{i=0}^{2}S_{i}}{3},T_{\min}),T_{\max})$$

$S_{i}$ presents the measured signal amplitude, i∈{0,1,2} denotes the baseline threshold, $T_{\max}$ denotes the maximum threshold, $T_{\min}$ denotes the minimum threshold, and α denotes the proportional coefficient.

(2) $$T_{t}=\beta \cdot T_{new}+(1-\beta )\cdot T_{t-1}$$

$T_{t}$ represents the updated adaptive threshold, $T_{t-1}$ denotes the threshold at the previous time step, and β denotes the smoothing factor.

After receiving the discrimination results, the processed data is transmitted via Bluetooth to the computer for signal observation and analysis.

#### 2.2.3. Communication Protocol

**Figure 6:**
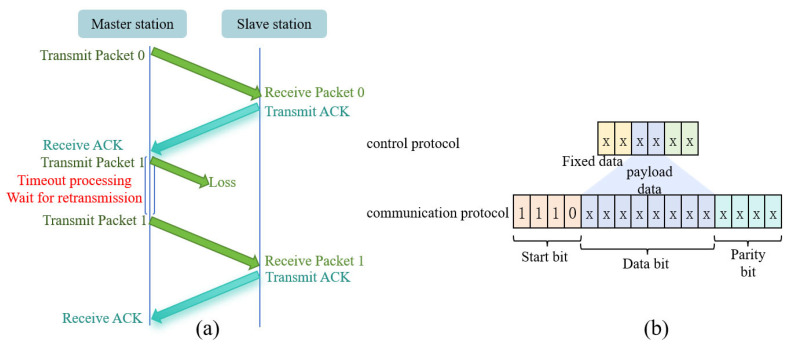
(**a**) ARQ protocol and (**b**) protocol framework.

The WARM system adopts a half-duplex communication protocol to prevent self-interference, accommodate complex underwater channel conditions, and ensure reliable data transmission. If the master station fails to receive a return signal within the designated timeframe, it retransmits the data to mitigate the losses caused by the channel variability. System performance is evaluated using the frame loss rate, providing a reliable basis for operation. The automatic repeat request process is illustrated in Figure 6a.

The system communication protocol includes a control protocol transmitted by the computer via Bluetooth and an acoustic transmission-based communication protocol. The control protocol frame contains three types of data: a fixed identifier, payload data, and an XOR checksum to ensure data integrity. In the underwater acoustic cation protocol, the sequence ‘1110’ functions as the start-of-frame to establish frame synchronization, thereby effectively reducing the interference. The protocol structure features a 4-bit start bit, 8-bit data bits, and a 4-bit checksum, ensuring transmission efficiency. Through the dual-layer protocol design, the computer can reliably send control commands, and the acoustic link guarantees the dependable transmission of the heart rate data, maintaining system communication robustness in complex underwater environments. The protocol framework is shown in Figure 6b.

### 2.3. Heart Rate Acquisition Module

The underwater heart rate monitoring system faces several technical challenges, including reliable heart rate signal acquisition, waterproof performance, and consistency of electrode adhesion.

**Figure 7:**
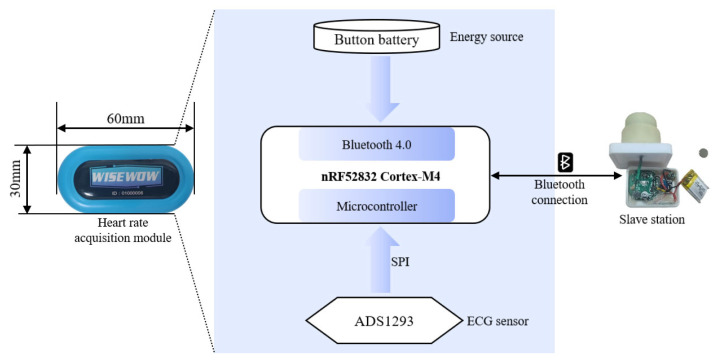
The whole framework of the heart rate acquisition module hardware design.

To address these requirements, heart rate was monitored by electrocardiography (ECG) via direct acquisition of the cardiac electrical activity. Stable signals and strong anti-interference capability were maintained even in underwater scenarios. Concurrently, the existing WISEWOW heart rate detection device was employed as the underwater monitoring hardware platform and was adjusted for underwater environments. The device primarily consists of an ECG acquisition circuit, a Bluetooth communication module, and a power supply module. It measures 60 mm × 30 mm and features an IP68 protection rating, with heart rate measurement accuracy reaching ±1 bpm. Figure 7 shows the key hardware components of the heart rate acquisition module. ECG signals were acquired by the heart rate sensor via conductive rubber electrodes embedded in a flexible chest strap. The signal is then digitized by the ADS1292, a high-resolution ADC chip. The converted digital signal is transmitted to the nRF51822 chip through the SPI interface. Finally, the microcontroller uses the Bluetooth 4.0 protocol to match the protocol of the slave station, thereby enabling real-time transmission of the heart rate data.

### 2.4. Ranging Design

**Figure 8:**
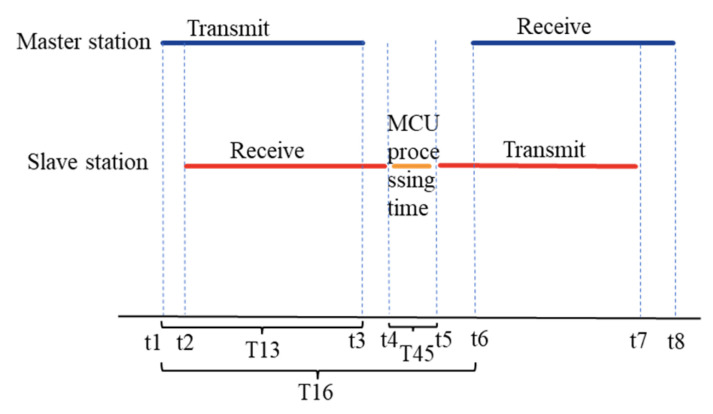
Timing diagram for distance measurement.

To ensure the safety of the swimmers during the training and competition, the system incorporates a ranging function in addition to the heart rate monitoring. The distance is estimated based on the time of the bidirectional acoustic communication between the master and slave stations. As shown in Figure 8, the time when the master station transmits the waveform is T13. When the slave station receives the signal, it performs short delay processing with the delay time T45, and then returns the delay time. After the master station receives the returned information, it records the current time T16. Thus, the underwater transmission time of the acoustic wave is Ts.

(3) Ts=T16−T45−T13

The propagation speed of acoustic waves in water is M, with M being 1500 m/s. The distance from the master station end to the slave station end is d.

(4) d=Ts/2.0*M

## 3. Experiments and Results

**Figure 9:**
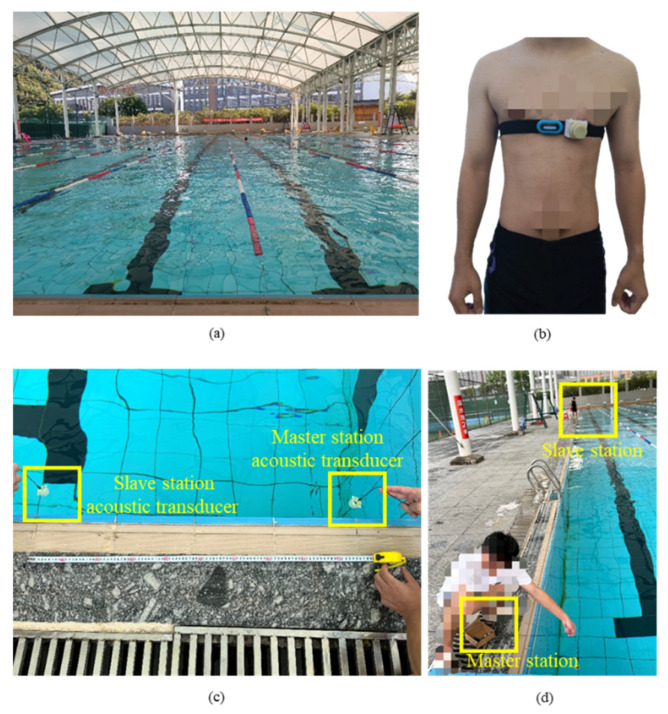
Experimental scenario diagram and real-life image of volunteers wearing devices for testing. (**a**) Test environment. (**b**) Subject wearing the system. (**c**) The 100 cm distance test. (**d**) The 2500 cm distance test.

This experiment aims to validate a wearable, amphibious acoustic-Bluetooth heart rate monitoring and ranging system. This experiment efficiently evaluates the system’s reliability, distance measuring, and real-time heart rate monitoring. Experiments were conducted in a standard swimming pool with a depth of 200 cm and dimensions of 5000 cm × 2500 cm. The master station was placed 50 cm from the pool wall and 50 cm below the water surface. In the frame loss rate (FLR) testing and ranging experiment, the master and slave stations were located at various distances—100, 500, 1000, 1500, 2000, and 2500 cm—each performing 180 communication sessions. The test environment is shown in Figure 9.

### 3.1. System Power Consumption Testing

**Figure 10:**
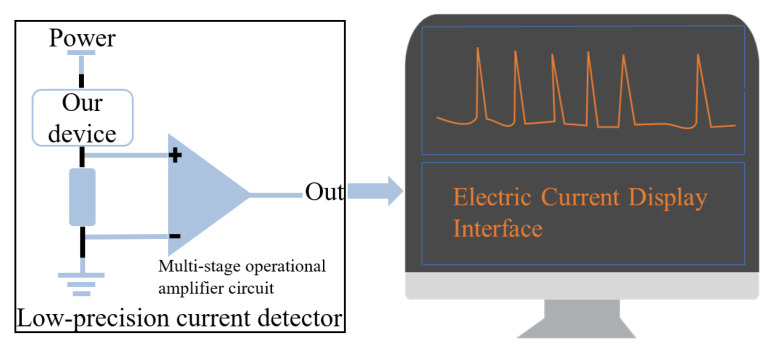
Wiring architecture diagram of the system under test and high-precision power analyzer.

**Figure 11:**
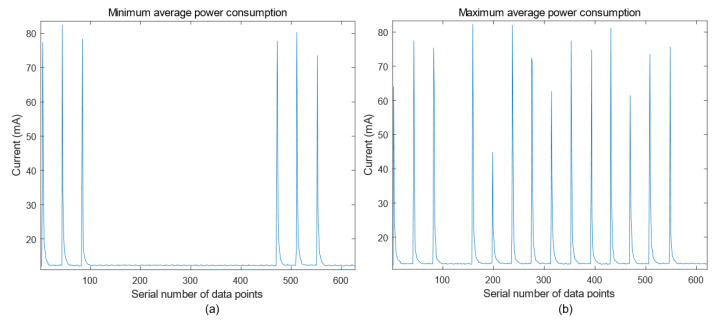
Current test diagram of the entire device. (**a**) Minimum power consumption plot during all-0 bit transmission; (**b**) Maximum power consumption plot during all-1 bit transmission.

The power consumption efficiency was evaluated using a microampere-level power analyzer, as shown in Figure 10. The lowest average power consumption occurred when all transmitted bits were zero, whereas the highest occurred when all bits were one. As shown in Figure 11, when the system operated at the lowest average power consumption, the peak instantaneous current reached 82.56 mA with an average current of 13.67 mA. When the system operated at the highest average power consumption, the peak instantaneous current was 82.26 mA with an average current of 15.24 mA. At the same time, the voltage amplitude remained at 3.8 V. Accordingly, the maximum instantaneous power consumption of the system was calculated to be 313.73 mW. The average power consumption during transmission ranged from 51.95 mW to 57.91 mW.

### 3.2. FLR Testing

**Figure 12:**
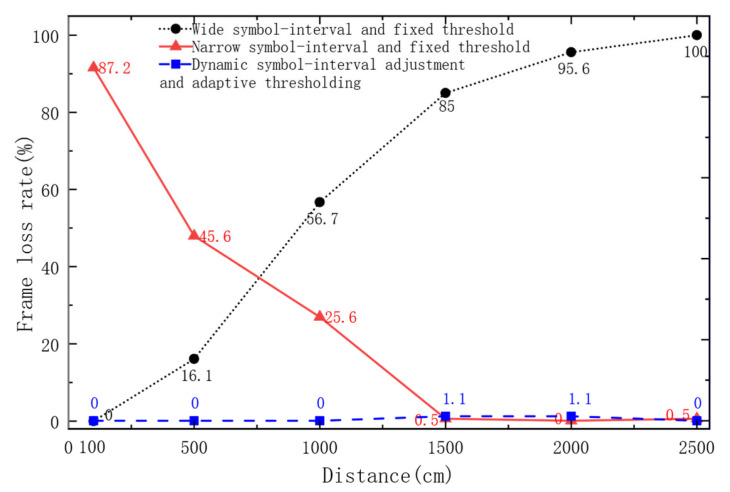
Comparison of frame loss rates over 180 transmissions under different communication conditions.

The FLR was used to verify the system’s reliability [23]. The results of the communication tests are shown in Figure 12.

The communication performance of the system was evaluated under different conditions and distances. Under the condition of a wide symbol interval and fixed threshold, an FLR of 0 was observed at 100 cm; thereafter, the FLR was found to gradually increase with distance, and by 2500 cm, the signal was completely lost. Under the condition of a narrow symbol interval and fixed threshold, a high FLR of 87.2% was recorded at 100 cm, but it was observed to decrease progressively as the distance increased, eventually dropping to 0.5% at 2500 cm. Under the condition of dynamic symbol-interval adjustment and adaptive thresholding, an FLR of 0 was maintained at 100, 500, 1000, and 2500 cm. Only at 1500 cm and 2000 cm were two packet losses, respectively, observed corresponding to an FLR of 1.1% at each of these distances. It was demonstrated by the comparative experiments that the FLR of the system was effectively reduced, and the communication efficiency was improved through the adoption of the dynamic symbol-interval adjustment and adaptive thresholding.

### 3.3. Amphibious Real-Time Heart Rate Monitoring and Validation Experiment

To validate the effectiveness of real-time heart rate monitoring, the reliability of the system was evaluated. A set of in-place jogging experiments was conducted, during which participants simultaneously wore two heart rate acquisition modules: the heart rate module used in this system and the Polar H10 [24], which served as the gold standard reference. The data were transmitted via underwater acoustic communication by the proposed device, while the Bluetooth transmission was used by the Polar H10, and the experimental data were then compared. In addition, an underwater real-time test was performed in the environment shown in Figure 9a, where the heart rate acquisition module and the slave station were worn by the subject. To comprehensively capture the heart rate monitoring data across diverse athletic scenarios, the experimental design encompasses three states: surface swimming, underwater swimming, and land-based activities. Consequently, the system incorporates breaststroke, underwater swimming, resting state, and water-land transition activities. The system collects and transmits the heart rate of swimming subjects at a frequency of 1 Hz.

Six subjects were recruited for the experiment, with an average age of 23 ± 3 years, body weight of 60 ± 10 kg, and height of 1.70 ± 0.1 m, as shown in Table 2. All participants had over two years of swimming experience and voluntarily signed informed consent forms.

**Figure 13:**
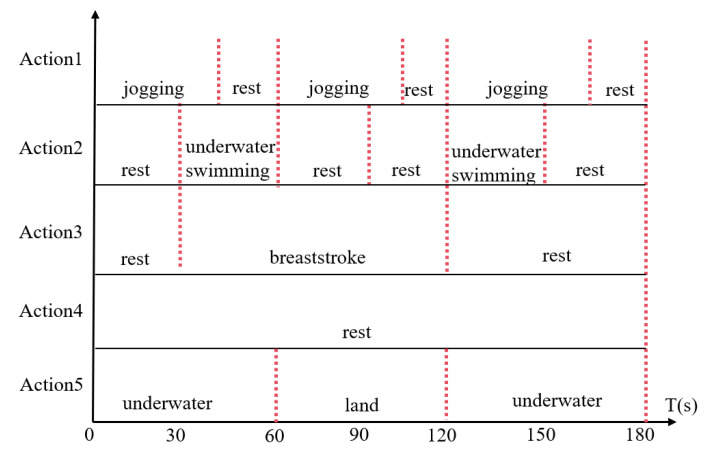
Timing diagram of the experiment.

The timeline of the experimental procedure is illustrated in Figure 13.

(1) Action 1: Three sets of jogging in place were performed, each set consisting of 40 s of jogging in place followed by 20 s of rest.
(2) Action 2: Two sets of underwater swimming were performed, with 30 s of resting in water, 30 s of swimming, and 30 s of rest.
(3) Action 3: Breaststroke swimming was performed, with 30 s of resting in water, 1.5 min of swimming, and 1 min of rest.
(4) Action 4: A resting state was maintained by the subject underwater for 3 min.
(5) Action 5: The subject was kept underwater for 1 min, then on land for 1 min, and subsequently underwater for another 1 min.

**Figure 14:**
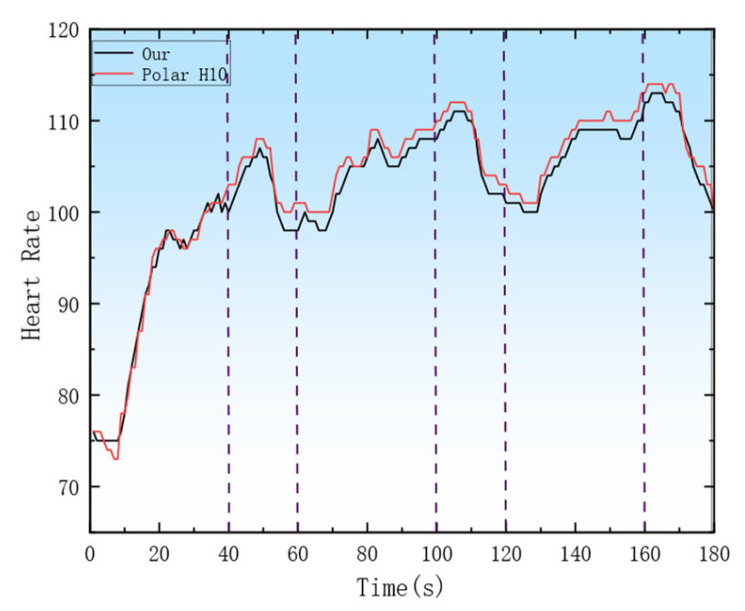
Comparison of heart rate data obtained from two heart rate acquisition modules simultaneously worn during jogging in place.

As shown in Figure 14, a rapid rise in heart rate was observed within the first 40 s of exercise. During the 40–60 s resting period, a pattern of an initial increase followed by a decline in heart rate was exhibited, and the same trend was observed in the subsequent two data sets. Meanwhile, a small heart rate error was observed between the data transmitted via underwater acoustic communication from the heart rate acquisition device and the measurements from the Polar H10, by which the effectiveness of the underwater acquisition system was effectively validated.

**Figure 15:**
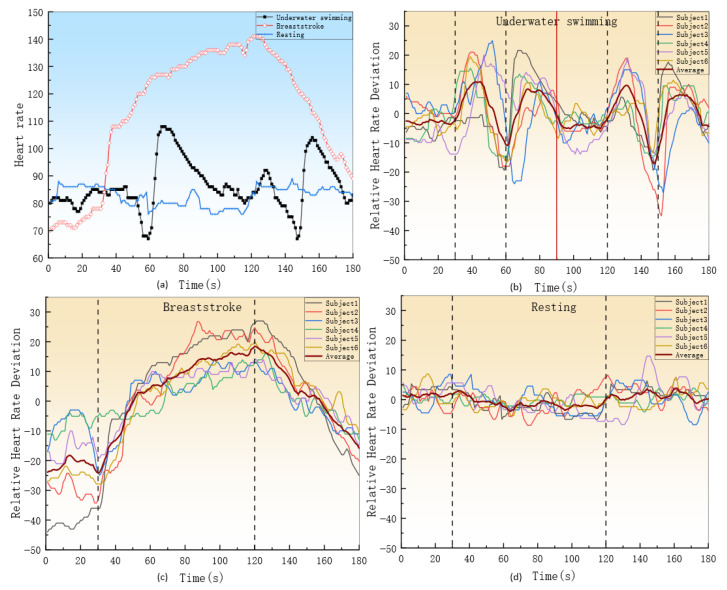
Heart rate of a swimmer under different swimming states and the relative heart rate deviation curves of six swimmers. (**a**) Heart rate curve; (**b**) diving; (**c**) breaststroke; (**d**) resting. The vertical dividing lines in the legend serve as time axis segmentation markers.

Figure 15a shows the heart rate curve of a swimmer. During the 30 s resting phase before the dive, the heart rate remains stable between 75 and 85 beats per minute (bpm). After the dive begins, the heart rate increases during the first 20 s but then drops to 67 bpm between 20 and 30 s. Upon surfacing to rest, the swimmer’s heart rate significantly increases before returning to the normal level. During the 30 s resting phase before the breaststroke, the heart rate remains stable. In the following 90 s breaststroke phase, heart rate showed an increase, rising by 30 to 70 beats per minute. During the 60 s rest period, heart rate gradually returns to normal. In the resting state, the heart rate remains within the normal range.

Figure 15b–d show the relative heart rate deviations of six swimmers during diving, breaststroke, and resting states, respectively. The relative deviations of the six swimmers were averaged.

**Figure 16:**
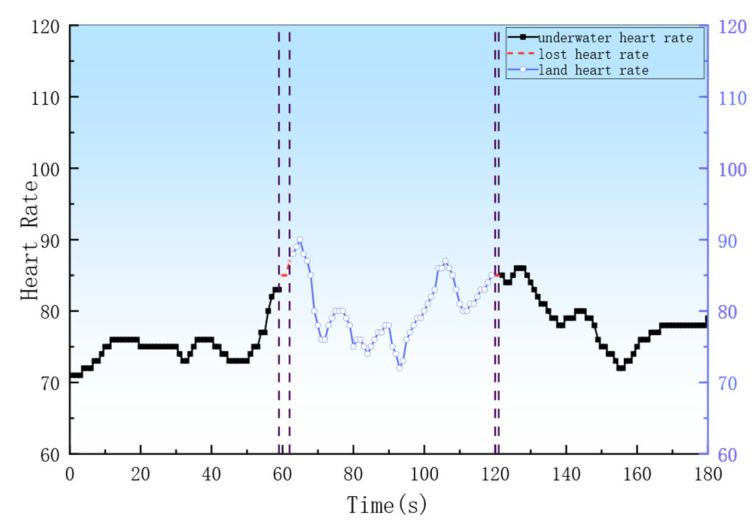
Amphibious heart rate monitoring chart. The vertical dividing lines in the legend serve as time axis segmentation markers.

As shown in Figure 16, the heart rate curve of the subject during the water-land transition reveals that during the 1 min underwater stay, the heart rate remained stable within the 70–90 beats per minute range. Subsequently, within the first minute after transitioning from underwater to land, the heart rate initially rose to 90 beats per minute before gradually declining to a stable level. Upon re-entering the water for a 1 min stay, the heart rate showed only a brief initial increase upon submersion before also returning to a stable state.

### 3.4. Ranging Testing

**Figure 17:**
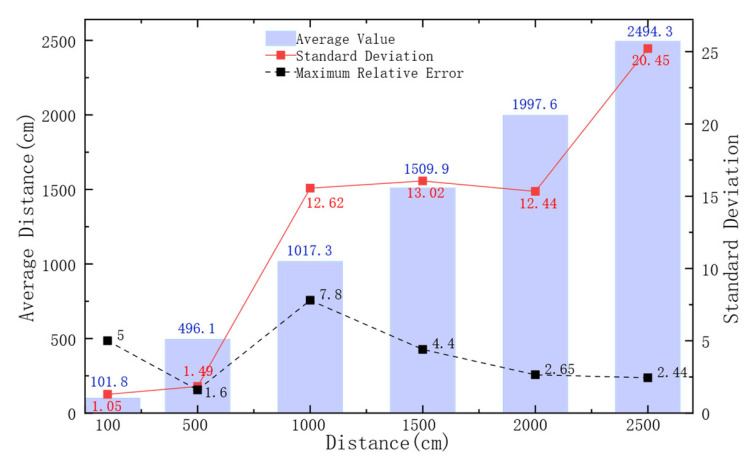
Proposed system’s ranging function test at different horizontal distances.

To verify the system’s distance ranging function, tests were conducted in the environment shown in Figure 9a. The results of the tests are shown in Figure 17.

Under different target distances, certain deviations were observed between the actual measured distances and the corresponding target distances. At a target distance of 100 cm, the smallest standard deviation and a relatively low mean error were obtained. At 500 cm, a standard deviation of 1.49 cm and a maximum relative error of 1.6% were recorded, with the data exhibiting good concentration. As the target distances increased to 1000, 1500, 2000, and 2500 cm, which was accompanied by slight fluctuations and increased dispersion in the data. Nevertheless, the maximum relative errors were still maintained at a low level, and the average measured values were kept close to the target distances, indicating high feasibility. Overall, across all tested distances, the average ranging errors of the system were all controlled below 50 cm, the error distribution was concentrated, and the maximum relative errors were generally small. As a result, the reliability of the distance measurement was demonstrated.

## 4. Discussion

This study developed and validated a wearable amphibious acoustic-Bluetooth heart rate monitoring and ranging system. The research team carried out several function tests on the system, confirming three research hypotheses: (1) the system offers wearability and low power consumption; (2) it enables real-time heart rate monitoring of amphibious swimmers; (3) it has achieved the range function.

### 4.1. System Power Consumption

In Experiment 1, the comparatively diminished currents at 198, 314, 469, etc., result from the insufficient charging and discharging capacity. The underwater acoustic propagation model in the swimming pools resembles that in the shallow seas. The transmission power and communication distance adhere to the $P=r^{1.5}$ model [25]. The minimum and maximum average power consumption of the system in transmission mode are both below 60 mW, which is considerably lower than that of existing underwater acoustic communication systems, thus validating the effectiveness of the proposed optimization scheme [12,21].

### 4.2. FLR Detection

In Experiment 2, under the condition of a wide symbol interval and a fixed threshold, the FLR was found to increase gradually with distance. This may be attributed to the originally small amplitude of the transmitted signal; at short distances, reliable demodulation could still be achieved, but as the distance increased, the signal amplitude was further attenuated and submerged in noise, and the fixed threshold became difficult to reach, thus leading to demodulation failure. Under the condition of a narrow symbol interval and a fixed threshold, the FLR was observed to decrease progressively as the distance increased. This phenomenon might be explained by the possibility that, at short distances, the signal amplitude was relatively large, and strong reflections caused signal saturation, which aggravated inter-symbol interference and consequently induced demodulation errors. As the distance increased, signal attenuation partially alleviated such saturation and interference, so the FLR was reduced, and the influence of the threshold itself was relatively limited in this situation. After the dynamic symbol-interval adjustment and adaptive thresholding condition were adopted, the signal width and the decoding threshold were automatically adjusted according to the symbol characteristics, and the decoding success rate was substantially improved. Nevertheless, two packet losses were still recorded at 1500 cm and at 2000 cm, respectively, which was likely caused by occasional multipath interference-induced decoding errors.

### 4.3. Heart Rate Detection

In Experiment 3, as shown in Figure 15a, the swimmer’s heart rate significantly increased due to engaging in aerobic activity at 90 s into the breaststroke, a heart rate of 140 beats per minute, and moderate-intensity exercise [26]. This finding is similar to that reported by Huntula, who observed a heart rate increase of 20–35 bpm during moderate-intensity exercise [27]. In Figure 16, the loss of heart rate data resulted due to the communication method switching during the transition between aquatic and terrestrial environments for heart rate. In Figure 15b, during the dive, the swimmer’s heart rate dropped between 20 and 30 s of breath-holding, which is consistent with the conclusion by Alentejano TC, who noted a significant heart rate reduction during breath-hold swimming at 20 and 25 s [28]. A more extensive comparison with the Polar H10 has not been pursued in this discussion; more comprehensive data and analyses will be reported in a separate forthcoming work. The experiment confirms the system’s effectiveness in reliably transmitting heart rate data and providing real-time monitoring.

### 4.4. Ranging System Evaluation

In Experiment 4, under short-distance conditions (100 cm and 500 cm), the propagation path of the acoustic signals was short, and the signal propagation delay was minimally affected by factors such as water absorption attenuation, multipath effects, and environmental disturbances. Consequently, optimal ranging performance was exhibited by the system in these two test groups, and high data concentration and repeatability were achieved. When the target distance was increased to 1000 cm, 1500 cm, 2000 cm, and 2500 cm, slight increases in the standard deviation and dispersion of the measured data were observed. This was likely because, at the medium-to-long distances, the propagation path of the acoustic signals was significantly lengthened, and the interference effects of water absorption attenuation, interface multipath reflections, and water inhomogeneity on the signals gradually accumulated, whereby small fluctuations in the measured values were inevitably introduced.

Real-time heart rate monitoring helps coaches develop personalized swimming plans and spot early signs of overtraining. When an athlete’s heart rate remains elevated and is difficult to normalize, it may indicate overexertion, suggesting the need to reduce exercise intensity to protect the swimmer’s health. Coaches can use this data to adjust training loads, lower injury risk, and promote proper recovery [29].

For the present study, the chest-strap heart rate acquisition structure was selected with underwater high-accuracy and high-reliability monitoring as the primary goal, and measurement accuracy was prioritized as the foremost criterion. In the chest-strap ECG approach, cardiac electrical activity was captured via electrodes positioned close to the heart. The obtained signals were characterized by stability, a high signal-to-noise ratio, and considerable immunity to motion artifacts. At the same time, measurement failure caused by the underwater attenuation of optical signals in wrist-worn PPG devices was effectively prevented [30].

Regarding wear comfort, the total weight of the device was reduced to 54 g by a lightweight design, and a highly elastic, flexible strap was employed. The subject tests confirmed that no interference with swimming movements was caused and no obvious discomfort was reported. The wristband-style structure was advantageous only in daily wear convenience, whereas it could not meet the core demands of the underwater dynamic monitoring in this research. By contrast, with the chest-strap structure, wearing impact was minimized while the measurement accuracy was guaranteed, and it was therefore adopted as the reasonable choice for the scenario under this study.

This study provides valuable theoretical and technical references for designing reliable, low-power, wearable underwater acoustic communication systems, highlighting their broad application potential. It establishes a basis and provides a reference value for subsequent study in swimming sports. Despite the several advantages offered by the system, a number of limitations remain in practical applications, including those related to communication range, transmission rate, and structural design. Moreover, tests have been conducted only in a controlled pool environment, and the application has not yet been extended to lakes or marine settings.

For further study, we intend to enhance the single-channel heart rate acquisition device to a multichannel collector that will capture information like blood oxygen levels, heart rate, electromyographic signals, and breathing rate. Additionally, we will implement integrated processing of hardware devices to support long-distance transmission, modify communication protocols, and explore more effective modulation and demodulation techniques for underwater communication to address multipath interference. It can also be applied to underwater swimmer positioning, speed measurement, and other related applications. We aim to combine AI technology with swimming training to facilitate scientific athletic development, consequently improving competitors’ performance. Simultaneously, we will integrate substantial AI algorithms to conduct real-time analyses of the swimmers’ data and forecast weariness and hazards [31].

## 5. Conclusions

This study introduces a wearable acoustic-Bluetooth dual model communication-based real-time heart rate monitoring and ranging system, primarily designed for underwater applications. This approach adopts a hardware-software co-design strategy to mitigate the limitations of conventional devices, including bulky size, high power consumption, and insufficient wearability. It provides a valuable reference for underwater motion monitoring research. The main contributions of this work are: (1) designing and implementing a wearable, low-power underwater acoustic communication system; (2) enabling real-time monitoring of swimmers’ heart rates; and (3) providing underwater ranging capability. Therefore, the safety of the swimmers can be guaranteed through the real-time heart rate monitoring and distance measurement provided by the proposed system.

## Figures and Tables

**Table 1:** Comparison of existing technique with proposed system.

Authors	Voltage (V)	Average Power Consumption (mW)	Distance (m)	Device Size	Advantages	Disadvantages
Jeon et al. [12]	14.8	2900	40	70 mm × 70 mm × 35 mm	Compact size; flexible node deployment	High power consumption
Won, T.-H et al. [13]	14.8	4500	70	70 mm × 70 mm × 35 mm	Compact size; compatibility with mobile devices; low cost	High power consumption
van Kleunen et al. [14]	11.1	8000	50–100	Not Reported *	Highly integrated system; localization capability	High power consumption; large device size
Mayberry et al. [15]	12–16.8	2400–30,000	50–220	Not Reported *	open-source design	High power consumption
Campagnaro et al. [16]	12	1120	100	Not Reported *	underwater localization	High power consumption; large device size
Bube et al. [20]	10–18	2400	200	50 mm × 50 mm × 150 mm	Wearable design; real-time communication	High power consumption
Xiao ZK et al. [21]	16	4800	100	Not Reported *	early-warning capability; high system integration	High power consumption; large device size
Proposed	3.8	52–58	25	47 mm × 36 mm × 18 mm	low power consumption; compact size; omnidirectional communication	Suitable for short- to medium-range communication

*: The reference does not report the exact dimensions, yet the system equipment can be seen to be substantially larger than that used in our experiments.

**Table 2:** Subject demographic information table.

Number	Gender	Age	Height (cm)	Weight (kg)
1	Male	24	167	83
2	Male	21	173	90
3	Male	25	172	57
4	Male	24	165	62.5
5	Male	24	177	68
6	Male	23	181	71

## Data Availability

The original contributions presented in this study are included in the article. Further inquiries can be directed to the corresponding author.
